# Supplementary material for: Water Stress Promotes Secondary Sexual Dimorphism in Ecophysiological Traits of Papaya Seedlings
Source: Plants (Basel). 2025 Aug 7;14(15):2445. doi: 10.3390/plants14152445 (PMC12349495; doi:10.3390/plants14152445)
Supplement: Supplementary file 1 [file plants-14-02445-s001.zip › plants-3724421-supplementary.pdf]

plants

MDPI

Supplementary Materials for:

Water Stress Promotes Secondary Sexual Dimorphism in Ecophysiological Traits of Papaya Seedlings

Ingrid Trancoso [1], Guilherme A.R. de Souza [1,2], João Vitor Paravidini de Souza [1,2], Rosana Maria dos Santos Nani de Miranda [1], Diesily de Andrade Neves [1], Miroslava Rakocevic [1*] and Eliemar Campostrini [1,2]

1 Setor de Fisiologia Vegetal, Laboratório de Melhoramento Genético Vegetal, Centro de Ciências e Tecnologias Agropecuárias, Universidade Estadual do Norte Fluminense, Avenida Alberto Lamego 2000, Parque Califórnia, Campos dos Goytacazes - RJ, 28013–602, Brazil; ingrid-trancoso@hotmail.com; guilherme.rodrigues@edu.uniube.br; nani@pq.uenf.br; diesilyandrade@gmail.com; mima.rakocevic61@gmail.com; campostenator@gmail.com

2 Department of Sustainable Crop Production, Università Cattolica del Sacro Cuore, Piacenza 29122, Italy; joaovitor.paravidini@unicatt.it

* Correspondence: mima.rakocevic61@gmail.com; Tel.: 55-19-97161-8918

Supplementary Table S1. Three-way ANOVA* of different mixed linear models (lme) adjustments for water (Wat), variety (Var), gender (Gen) and their interaction effects during four days (1st to 4th) after the water-shortage interruption on leaf stomatal conductance (g_s), SPAD index, greenness index, combination of normalized difference vegetation index (CNDVI), Carotenoid reflectance index 1 (CRI1), carotenoid reflectance index 2 (CRI2), flavonoid reflectance index (FRI), photochemical reflectance index (PRI), water band index (WBI), structure intensive pigment index (SIPI), simple ratio pigment index (SRPI), leaf number (LN), basal stem diameter (SD), leaf dry mass (LDM), stem dry mass (SDM), root dry mass (RDM), total dry mass (TDM), leaf mass ratio (LMR), and root to shoot ratio (RShR). Identification of the figure from the manuscript where the data are shown is indicated at a left table side.

	Vari-able	Day after WS inter.	NumDF (DenDF) Wat	NumDF (DenDF) Var	NumDF (DenDF) Gen	NumDF (DenDF) Wat x Var	NumDF (DenDF) Wat x Gen	NumDF (DenDF) Var x Gen	NumDF (DenDF) Wat x Var x Gen	F_{Wat} (P_{Wat})	F_{Var} (P_{Var})	F_{Gen} (P_{Gen})	$F_{\text{Wat x Var}}$ ($P_{\text{Wat x Var}}$)	$F_{\text{Wat x Gen}}$ ($P_{\text{Wat x Gen}}$)	$F_{\text{Var x Gen}}$ ($P_{\text{Var x Gen}}$)	$F_{\text{Wat x Var x Gen}}$ ($P_{\text{Wat x Var x Gen}}$)
Figure 1	g_s	1st	1<br>(134)	1<br>(134)	1<br>(134)	1<br>(134)	1<br>(134)	1<br>(134)	-<br>-	3.9070<br>(0.0501)	0.6627<br>(0.4170)	2.7359<br>(0.1005)	**23.2662**<br>**(<.0001)**	0.1280<br>(0.7210)	**5.2569**<br>**(0.0234)**	-<br>-
		2nd	1<br>(136)	1<br>(136)	1<br>(136)	1<br>(136)	-<br>-	-<br>-	-<br>-	1.0904<br>(0.2982)	0.6729<br>(0.4135)	2.7259<br>(0.1010)	**11.7507**<br>**(0.0008)**	-<br>-	-<br>-	-<br>-
		3rd	1<br>(136)	1<br>(136)	1<br>(136)	1<br>(136)	-<br>-	-<br>-	-<br>-	0.0117<br>(0.9137)	3.2198<br>(0.0750)	0.3000<br>(0.5848)	**16.9404**<br>**(0.0001)**	-<br>-	-<br>-	-<br>-
		4th	1<br>(136)	1<br>(136)	1<br>(136)	1<br>(136)	-<br>-	-<br>-	-<br>-	0.0013<br>(0.9708)	0.3142<br>(0.5760)	0.4793<br>(0.4899)	**6.5197**<br>**(0.0118)**	-<br>-	-<br>-	-<br>-
Figure 2A	SPAD	1st	1<br>(136)	1<br>(136)	1<br>(136)	1<br>(136)	-<br>-	-<br>-	-<br>-	**9.4088**<br>**(0.0026)**	**5.3016**<br>**(0.0228)**	0.0026<br>(0.9597)	**5.6135**<br>**(0.0192)**	-<br>-	-<br>-	-<br>-
		2nd	1<br>(136)	1<br>(136)	1<br>(136)	1<br>(136)	-<br>-	-<br>-	-<br>-	**13.7647**<br>**(0.0003)**	**4.9735**<br>**(0.0274)**	0.0797<br>(0.7781)	**5.0615**<br>**(0.0261)**	-<br>-	-<br>-	-<br>-
		3rd	1<br>(136)	1<br>(136)	1<br>(136)	1<br>(136)	-<br>-	-<br>-	-<br>-	**11.1773**<br>**(0.0011)**	**6.6431**<br>**(0.0110)**	0.0246<br>(0.8757)	**4.6479**<br>**(0.0329)**	-<br>-	-<br>-	-<br>-
		4th	1	1	1	1	1	1	1	0.3442	**5.8916**	1.6747	0.1306	**6.7537**	0.5667	**3.9209**

			(133)	(133)	(133)	(133)	(133)	(133)	(133)	(0.5584)	**(0.0166)**	(0.1979)	(0.7184)	**(0.0104)**	(0.4529)	**(0.0498)**
Figure 2B	Gidex	1st	1<br>(137)	1<br>(137)	1<br>(137)	-<br>-	-<br>-	-<br>-	-<br>-	0.6340<br>(0.4274)	**45.2500<br>(<.0001)**	0.6630<br>(0.4167)	-<br>-	-<br>-	-<br>-	-<br>-
		2nd	1<br>(136)	1<br>(136)	1<br>(136)	1<br>(136)	-<br>-	-<br>-	-<br>-	**16.5105<br>(0.0001)**	**5.7229<br>(0.0196)**	0.1289<br>(0.7207)	**18.2036<br>(0.0001)**	-<br>-	-<br>-	-<br>-
		3rd	1<br>(133)	1<br>(133)	1<br>(133)	1<br>(133)	1<br>(133)	1<br>(133)	1<br>(133)	**20.5860<br>(<.0001)**	3.7131<br>(0.0561)	1.8491<br>(0.1762)	**16.0458<br>(0.0001)**	**11.5078<br>(0.0009)**	0.7624<br>(0.3842)	**11.4336<br>(0.0009)**
		4th	1<br>(135)	1<br>(135)	1<br>(135)	1<br>(135)	1<br>(135)	-<br>-	-<br>-	0.5915<br>(0.4432)	**2.5511<br>(0.1126)**	2.0742<br>(0.1521)	**4.9889<br>(0.0272)**	**5.9309<br>(0.0162)**	-<br>-	-<br>-
Figure 2C	CNDVI	1st	1<br>(136)	1<br>(136)	1<br>(136)	1<br>(136)	-<br>-	-<br>-	-<br>-	**11.9986<br>(0.0007)**	0.0165<br>(0.8980)	0.0027<br>(0.9588)	**5.1601<br>(0.0247)**	-<br>-	-<br>-	-<br>-
		2nd	1<br>(135)	1<br>(135)	1<br>(135)	1<br>(135)	1<br>(135)	-<br>-	-<br>-	**5.5421<br>(0.0216)**	0.0700<br>(0.7921)	1.7138<br>(0.1951)	**22.2222<br>(<.0001)**	**5.1200<br>(0.0270)**	-<br>-	-<br>-
		3rd	1<br>(136)	1<br>(136)	1<br>(136)	1<br>(136)	-<br>-	-<br>-	-<br>-	3.6379<br>(0.0586)	**4.1390<br>(0.0438)**	**5.3556<br>(0.0222)**	**5.7131<br>(0.0182)**	-<br>-	-<br>-	-<br>-
		4th	1<br>(135)	1<br>(135)	1<br>(135)	1<br>(135)	1<br>(135)	-<br>-	-<br>-	**4.117<br>(0.0444)**	0.1920<br>(0.6618)	2.8780<br>(0.0921)	**3.0750<br>(0.0418)**	**3.9580<br>(0.0487)**	-<br>-	-<br>-
Figure 3A	CRI1	1st	1<br>(137)	1<br>(137)	1<br>(137)	-<br>-	-<br>-	-<br>-	-<br>-	**42.1600<br>(<.0001)**	0.7666<br>(0.3828)	0.3052<br>(0.5816)	-<br>-	-<br>-	-<br>-	-<br>-
		2nd	1<br>(137)	1<br>(137)	1<br>(137)	-<br>-	-<br>-	-<br>-	-<br>-	**2.7885<br>(0.0496)**	**16.6090<br>(0.0001)**	2.0616<br>(0.1557)	-<br>-	-<br>-	-<br>-	-<br>-
		3rd	1<br>(137)	1<br>(137)	1<br>(137)	-<br>-	-<br>-	-<br>-	-<br>-	0.4287<br>(0.5137)	**38.0620<br>(<.0001)**	0.0153<br>(0.9017)	-<br>-	-<br>-	-<br>-	-<br>-

		4th	1<br>(134)	1<br>(134)	1<br>(134)	1<br>(134)	1<br>(134)	1<br>(134)	-<br>-	**7.9448**<br>**(0.0056)**	**28.7301**<br>**(<.0001)**	**15.4224**<br>**(0.0001)**	1.0524<br>(0.3068)	2.9136<br>(0.0902)	**11.6745**<br>**(0.0008)**	-<br>-
Figure 3B	CRI2	1st	1<br>(137)	1<br>(137)	1<br>(137)	-<br>-	-<br>-	-<br>-	-<br>-	**52.2297**<br>**(<.0001)**	**19.1704**<br>**(<.0001)**	0.0110<br>(0.9167)	-<br>-	-<br>-	-<br>-	-<br>-
		2nd	1<br>(136)	1<br>(136)	1<br>(136)	1<br>(136)	-<br>-	-<br>-	-<br>-	**8.0708**<br>**(0.0060)**	0.8387<br>(0.3631)	2.5211<br>(0.1171)	**9.4470**<br>**(0.0031)**	-<br>-	-<br>-	-<br>-
		3rd	1<br>(135)	1<br>(135)	1<br>(135)	1<br>(135)	1<br>(135)	-<br>-	-<br>-	0.0059<br>(0.9388)	**12.5379**<br>**(0.0005)**	2.3878<br>(0.1246)	0.0138<br>(0.9068)	**5.2913**<br>**(0.0230)**	-<br>-	-<br>-
		4th	1<br>(134)	1<br>(134)	1<br>(134)	1<br>(134)	1<br>(134)	1<br>(134)	-<br>-	**7.7472**<br>**(0.0062)**	**20.8955**<br>**(<.0001)**	**16.4180**<br>**(0.0001)**	2.2333<br>(0.1374)	**12.1304**<br>**(0.0007)**	**6.5829**<br>**(0.0114)**	-<br>-
Figure 3C	FRI	1st	1<br>(136)	1<br>(136)	1<br>(136)	1<br>(136)	-<br>-	-<br>-	-<br>-	0.8240<br>(0.3658)	**3.9680**<br>**(0.0484)**	0.5990<br>(0.4404)	**4.1340**<br>**(0.0440)**	-<br>-	-<br>-	-<br>-
		2nd	1<br>(137)	1<br>(137)	1<br>(137)	-<br>-	-<br>-	-<br>-	-<br>-	**5.1490**<br>**(0.0265)**	1.7011<br>(0.1966)	1.0376<br>(0.3120)	-<br>-	-<br>-	-<br>-	-<br>-
		3rd	1<br>(137)	1<br>(137)	1<br>(137)	-<br>-	-<br>-	-<br>-	-<br>-	**15.1152**<br>**(0.0002)**	**21.2633**<br>**(<.0001)**	2.0535<br>(0.1541)	-<br>-	-<br>-	-<br>-	-<br>-
		4th	1<br>(137)	1<br>(137)	1<br>(137)	-<br>-	-<br>-	-<br>-	-<br>-	**15.8412**<br>**(0.0001)**	0.2176<br>(0.6416)	0.1162<br>(0.7337)	-<br>-	-<br>-	-<br>-	-<br>-
Figure 4A	PRI	1st	1<br>(133)	1<br>(133)	1<br>(133)	1<br>(133)	1<br>(133)	1<br>(133)	1<br>(133)	**0.0484**<br>**(0.8262)**	3.6149<br>(0.0594)	0.8344<br>(0.3626)	0.1010<br>(0.7512)	**4.0001**<br>**(0.0475)**	0.3398<br>(0.5609)	**4.7069**<br>**(0.0318)**
		2nd	1<br>(136)	1<br>(136)	1<br>(136)	1<br>(136)	-<br>-	-<br>-	-<br>-	**9.5835**<br>**(0.0029)**	0.2228<br>(0.6385)	0.0416<br>(0.8390)	**12.7639**<br>**(0.0007)**	-<br>-	-<br>-	-<br>-
		3rd	1<br>(135)	1<br>(135)	1<br>(135)	1<br>(135)	1<br>(135)	-<br>-	-<br>-	**10.2723**<br>**(0.0017)**	0.1680<br>(0.6826)	0.0001<br>(0.9913)	2.7366<br>(0.1004)	**4.5911**<br>**(0.0339)**	-<br>-	-<br>-

		4th	1<br>(135)	1<br>(135)	1<br>(135)	1<br>(135)	1<br>(135)	-<br>-	-<br>-	2.7612<br>(0.0989)	0.0217<br>(0.8831)	2.2841<br>(0.1330)	**12.0900**<br>**(0.0007)**	**8.9346**<br>**(0.0033)**	-<br>-	-<br>-
Figure 4B	WBI	1st	1<br>(137)	1<br>(137)	1<br>(137)	-<br>-	-<br>-	-<br>-	-<br>-	**45.6780**<br>**(<.0001)**	**7.3890**<br>**(0.0074)**	0.0900<br>(0.7649)	-<br>-	-<br>-	-<br>-	-<br>-
		2nd	1<br>(137)	1<br>(137)	1<br>(137)	-<br>-	-<br>-	-<br>-	-<br>-	0.3980<br>(0.5302)	2.9210<br>(0.0921)	0.6130<br>(0.4363)	-<br>-	-<br>-	-<br>-	-<br>-
		3rd	1<br>(137)	1<br>(137)	1<br>(137)	-<br>-	-<br>-	-<br>-	-<br>-	0.6630<br>(0.4171)	0.9040<br>(0.3434)	1.3820<br>(0.2419)	-<br>-	-<br>-	-<br>-	-<br>-
		4th	1<br>(136)	1<br>(136)	1<br>(136)	1<br>(136)	-<br>-	-<br>-	-<br>-	**31.1410**<br>**(<.0001)**	**9.5720**<br>**(0.0024)**	**5.8640**<br>**(0.0168)**	**13.2070**<br>**(0.0004)**	-<br>-	-<br>-	-<br>-
Figure 5A	SIPI	1st	1<br>(136)	1<br>(136)	1<br>(136)	1<br>(136)	-<br>-	-<br>-	-<br>-	24.1800<br>**(<.0001)**	0.8600<br>(0.3554)	0.3500<br>(0.5535)	**4.0300**<br>**(0.0468)**	-<br>-	-<br>-	-<br>-
		2nd	1<br>(136)	1<br>(136)	1<br>(136)	1<br>(136)	-<br>-	-<br>-	-<br>-	1.5800<br>(0.2133)	**6.0700**<br>**(0.0164)**	0.5900<br>(0.4447)	**9.2600**<br>**(0.0034)**	-<br>-	-<br>-	-<br>-
		3rd	1<br>(133)	1<br>(133)	1<br>(133)	1<br>(133)	1<br>(133)	1<br>(133)	1<br>(133)	0.0180<br>(0.8949)	3.0430<br>(0.0834)	**4.6530**<br>**(0.0328)**	0.0100<br>(0.9193)	**13.2440**<br>**(0.0004)**	**5.9030**<br>**(0.0164)**	**14.2610**<br>**(0.0002)**
		4th	1<br>(134)	1<br>(134)	1<br>(134)	1<br>(134)	1<br>(134)	1<br>(134)	-<br>-	**8.2600**<br>**(0.0047)**	**11.9100**<br>**(0.0007)**	**5.1900**<br>**(0.0243)**	2.4000<br>(0.1240)	0.0800<br>(0.7714)	**5.7200**<br>**(0.0182)**	-<br>-
Figure 5B	SRP	1st	1<br>(136)	1<br>(136)	1<br>(136)	1<br>(136)	-<br>-	-<br>-	-<br>-	3.1050<br>(0.0803)	**31.3790**<br>**(<.0001)**	2.4740<br>(0.1181)	**6.7690**<br>**(0.0103)**	-<br>-	-<br>-	-<br>-
		2nd	1<br>(137)	1<br>(137)	1<br>(137)	-<br>-	-<br>-	-<br>-	-<br>-	1.5200<br>(0.2220)	1.8090<br>(0.1831)	**4.9270**<br>**(0.0298)**	-<br>-	-<br>-	-<br>-	-<br>-
		3rd	1<br>(137)	1<br>(137)	1<br>(137)	-<br>-	-<br>-	-<br>-	-<br>-	0.1260<br>(0.7236)	1.6360<br>(0.2031)	0.0020<br>(0.9652)	-<br>-	-<br>-	-<br>-	-<br>-

	I	4th	1<br>(136)	1<br>(136)	1<br>(136)	1<br>(136)	-<br>-	-<br>-	-<br>-	**7.6550**<br>**(0.0064)**	0.0100<br>(0.9185)	0.0470<br>(0.8293)	-<br>-	-<br>-	-<br>-	-<br>-
6A	LN	4th	1<br>(135)	1<br>(135)	1<br>(135)	1<br>(135)	1<br>(135)	-<br>-	-<br>-	0.1311<br>(0.7178)	**12.5894**<br>**(0.0005)**	0.0642<br>(0.8003)	**5.0181**<br>**(0.0267)**	**8.7435**<br>**(0.0037)**	-<br>-	-<br>-
6B	SD	4th	1<br>(137)	1<br>(137)	1<br>(137)	-<br>-	-<br>-	-<br>-	-<br>-	**6.6830**<br>**(0.0108)**	**13.9376**<br>**(0.0003)**	01470<br>(0.7020)	-<br>-	-<br>-	-<br>-	-<br>-
6C	LDM	4th	1<br>(136)	1<br>(136)	1<br>(136)	1<br>(136)	-<br>-	-<br>-	-<br>-	**10.6246**<br>**(0.0014)**	**5.1842**<br>**(0.0244)**	**11.4885**<br>**(0.0009)**	**3.1065**<br>**(0.0282)**	-<br>-	-<br>-	-<br>-
6D	SDM	4th	1<br>(137)	1<br>(137)	1<br>(137)	-<br>-	-<br>-	-<br>-	-<br>-	**11.5011**<br>**(0.0009)**	0.3967<br>(0.5298)	0.0202<br>(0.8873)	-<br>-	-<br>-	-<br>-	-<br>-
6E	RDM	4th	1<br>(137)	1<br>(137)	1<br>(137)	-<br>-	-<br>-	-<br>-	-<br>-	0.0774<br>(0.7813)	0.4484<br>(0.5042)	1.4035<br>(0.2382)	-<br>-	-<br>-	-<br>-	-<br>-
6F	TDM	4th	1<br>(133)	1<br>(133)	1<br>(133)	1<br>(133)	1<br>(133)	1<br>(133)	1<br>(133)	0.3299<br>(0.5667)	2.8818<br>(0.0919)	0.0015<br>(0.9695)	**8.8169**<br>**(0.0035)**	**4.9696**<br>**(0.0275)**	0.4461<br>(0.5054)	**4.7082**<br>**(0.0318)**
6G	LMR	4th	1<br>(135)	1<br>(135)	1<br>(135)	1<br>(135)	1<br>(135)	-<br>-	-<br>-	0.0012<br>(0.9719)	0.1653<br>(0.6850)	0.0913<br>(0.7630)	1.1676<br>(0.2818)	**4.8246**<br>**(0.0298)**	-<br>-	-<br>-
6H	RShR	4th	1<br>(137)	1<br>(137)	1<br>(137)	-<br>-	-<br>-	-<br>-	-<br>-	**5.1382**<br>**(0.0250)**	1.6723<br>(0.1981)	0.0644<br>(0.8000)	-<br>-	-<br>-	-<br>-	-<br>-

* Confidence level was 0.95. *P*-value adjustment by Tukey method, with significance level of <0.05, when significant marked in bold.
